# Guarana (*Paullinia cupana*) Extract Protects *Caenorhabditis elegans* Models for Alzheimer Disease and Huntington Disease through Activation of Antioxidant and Protein Degradation Pathways

**DOI:** 10.1155/2018/9241308

**Published:** 2018-07-04

**Authors:** Patrícia Ferreira Boasquívis, Giovanna Melo Martins Silva, Franciny Aparecida Paiva, Rodrigo Marinho Cavalcanti, Cecília Verônica Nunez, Riva de Paula Oliveira

**Affiliations:** ^1^Núcleo de Pesquisa em Ciências Biológicas, Universidade Federal de Ouro Preto, Ouro Preto, MG, Brazil; ^2^Departamento de Biologia Celular e Genética, Universidade Federal do Rio Grande do Norte, Natal, RN, Brazil; ^3^Laboratório de Bioprospecção e Biotecnologia, Instituto Nacional de Pesquisas da Amazônia, Manaus, AM, Brazil; ^4^Departamento de Biodiversidade, Evolução e Meio Ambiente, Universidade Federal de Ouro Preto, Ouro Preto, MG, Brazil

## Abstract

Guarana (*Paullinia cupana*) is largely consumed in Brazil in high energy drinks and dietary supplements because of its stimulant activity on the central nervous system. Although previous studies have indicated that guarana has some protective effects in Parkinson's (PD), Alzheimer's (AD), and Huntington's (HD) disease models, the underlying mechanisms are unknown. Here, we investigated the protective effects of guarana hydroalcoholic extract (GHE) in *Caenorhabditis elegans* models of HD and AD. GHE reduced polyglutamine (polyQ) protein aggregation in the muscle and also reduced polyQ-mediated neuronal death in ASH sensory neurons and delayed *β*-amyloid-induced paralysis in a caffeine-independent manner. Moreover, GHE's protective effects were not mediated by caloric restriction, antimicrobial effects, or development and reproduction impairment. Inactivation of the transcription factors SKN-1 and DAF-16 by RNAi partially blocked the protective effects of GHE treatment in the AD model. We show that the protective effect of GHE is associated with antioxidant activity and modulation of proteostasis, since it increased the lifespan and proteasome activity, reduced intracellular ROS and the accumulation of autophagosomes, and increased the expression of SOD-3 and HSP-16.2. Our findings suggest that GHE has therapeutic potential in combating age-related diseases associated with protein misfolding and accumulation.

## 1. Introduction

Guarana is the popular name of *Paullinia cupana* var. *sorbilis* (Mart.) from which the seeds are widely used. In Brazil, guarana (ground seeds or extracts from seeds) is largely consumed in traditional medicine as well as in high energy drinks and dietary supplements, mostly due to its stimulant activity on the central nervous system (CNS) [1–3]. The stimulant effect of guarana is associated with high caffeine content, a psychoactive pseudoalkaloid well known for its benefits to human lifespan and aging-associated neuropathologies [4]. Guarana also contains other methylxanthines, including theophylline and theobromine, as well as polyphenols, such as catechins and epicatechins [5, 6]. This phytochemical composition has been associated with guarana's biological activities, which include antioxidant [7–9], antimicrobial [6], and chemoprophylactic activities in carcinogenesis [10, 11] and antigenotoxic effects [12].

Previous studies have evaluated guarana's effects on models of neurodegeneration and neurodegenerative disorders. Guarana extract prepared by extraction with DMSO showed a protective effect in human dopaminergic neuroblastoma SH-SY5Y cells exposed to rotenone, which is commonly used as an *in vitro* model for Parkinson's disease (PD) [13]. Bittencourt et al. [5] demonstrated that guarana powder prevents amyloid-*β* peptide (A*β*) aggregation *in vitro*, prevents protein glycation, and reduces oxidative stress and cellular death induced by acrolein, suggesting a beneficial effect for Alzheimer's disease (AD). Guarana powder prevented memory impairment and the decrease in acetylcholinesterase (AChE) activity in the hippocampus of hyperlipidemic rats, implying a beneficial effect of guarana on cognitive disorders [14]. Most recently, Peixoto et al. [15] found that guarana's water extract has antioxidant and antiaging effects in *Caenorhabditis elegans* models. Specifically, they showed that stress resistance induced by guarana is dependent on the transcription factor DAF-16, ortholog of FoxO proteins in mammals. Additionally, guarana water extracts reduced the formation of polyglutamine (polyQ) aggregates expressed in *C. elegans* muscle, suggesting a protective effect of guarana in Huntington's disease (HD) [15].

Despite the recent evidence showing the potential neuroprotective effects of guarana, little is known regarding its underlying mechanisms. The nematode *Caenorhabditis elegans* (*C. elegans*) is a valuable model for understanding the molecular mechanisms that modulate stress responses, aging, and neurodegenerative disorders. Several aggregation-prone human proteins associated with neurodegenerative diseases have been expressed in a variety of *C. elegans* tissues to understand protein misfolding and aggregation [16–18].

*C. elegans* has highly conserved transcription factors regulating stress resistance responses, longevity, and protein homeostasis, allowing for the elucidation of their role in protein toxicity and neurodegeneration [16, 17]. For example, the induction of heat shock transcription factor 1 (HSF-1/HSF1) was associated with a decrease in A*β* aggregation and toxicity in *C. elegans* models of AD [19]. Additionally, HSF-1/HSF1 knockdown increased polyQ protein aggregation in *C. elegans* models of HD [20]. Increased DAF-16/FoxO signaling reduced A*β*-associated phenotypes [21], suggesting a correlation between aging and A*β* toxicity [17]. Likewise, reducing protein aggregates by silencing *age-1*, in a DAF-16-dependent manner, demonstrated the correlation between aging-associated pathways and polyQ aggregation [22]. Also, overexpression of HSP-16.2, a chaperonin associated with HSF-1 and DAF-16, reduced A*β* toxicity [19, 23], and silencing HSP-70 increased protein aggregation in polyQ models [20]. Conversely, potential AD therapies, such as coffee, rich in caffeine, were shown to be dependent on SKN-1/Nrf2, a transcription factor associated with oxidative stress responses [24]. Moreover, the coffee treatment increased the expression of GST-4, a detoxification enzyme whose transcription is under the control of SKN-1/Nrf2 [24].

Here, we investigated the protective effects of guarana hydroalcoholic extract (GHE) in *C. elegans* models of HD and AD. We observed that GHE has a protective effect in both models, exhibited by delayed or reduced toxic phenotypes associated with protein misfolding and accumulation. Inactivation of *skn-1* and *daf-16* by RNAi blocked the protective effects of 10 mg/mL GHE treatment in the AD model, indicating that the transcription factors SKN-1 and DAF-16 are involved in GHE protection. Analysis with GFP (green fluorescent protein) reporter strains revealed that GHE treatment increased the expression of proteins associated with protein homeostasis, lysosome degradation, and oxidative stress responses. Biochemical analysis showed that GHE increases proteasome activity and reduces intracellular reactive oxygen species (ROS). Further, in the AD model, GHE delayed A*β*-induced paralysis in a caffeine-independent manner. Taken together, these results suggest that GHE alleviates polyQ protein aggregation and A*β*-induced toxicity by activating protein degradation and antioxidant defenses, partially through SKN-1 and DAF-16.

## 2. Material and Methods

### 2.1. Chemicals, Reagents, and Strains

Guarana powder (batch PGU00977) was purchased from Deg Fragon (Brazil). Methanol (MeOH), 2,2-diphenyl-1-picrylhydrazyl (DPPH), 6-hydroxy-2,5,7,8-tetramethylchroman-2-carboxylic acid (Trolox), Folin-Ciocalteu (FC) reagent, gallic acid, *tert-*butyl hydroperoxide (TBHP), fluorodeoxyuridine (FUDR), and 2,7-dichlorodihydrofluorescein diacetate (H_2_DCFDA) and the reference standards for caffeine, theobromine, catechin, and epicatechin (97–99.9% purity) were purchased from Sigma-Aldrich (St. Louis, MO, USA). Hydrogen peroxide (H_2_O_2_) was purchased from VETEC (Duque de Caxias, Rio de Janeiro, Brazil).

The following *Caenorhabditis elegans* strains were used: N2 (*wild-type strain*); CL4176 (*smg-1(cc546)*; *dvls27[pAF29(myo-3/Aβ1–42/letUTR)]* + *pRF4[rol-6(su10069)*); CL2006 (*dvIs2[pCL12(unc-54/human Abeta peptide 1–42 minigene) + pRF4]*); AM141 (*rmIs133 [unc-54p::Q40::YFP]*); HA759 (*pqe-1(rt13)*; *rtIs11 [osm-10p::GFP + osm-10p::HtnQ150 + dpy-20(+)]*); TJ375 (*gpls[hsp-16.2::GFP]*); CF1553 (*muIs84 [pAD76(sod-3::GFP)]*); and CL2166 (*dvls19[pAF15(gst-4::GFP::NLS)]*).

### 2.2. Guarana Hydroalcoholic Extract (GHE) Preparation

GHE was prepared according to Bittencourt et al. [7]. Briefly, guarana powder was extracted by diluting with ethanol : water (70 : 30, *v*/*v*) at a plant/solvent ratio 300 mg/1 mL and stirred for 24 h. Next, the suspension was centrifuged at 3000 rpm for 10 min and the solvent was removed from the resulting liquid extract (supernatant) by rotary evaporation. GHE was kept in a ventilated oven at 50°C for approximately 24 h until the solvent was completely removed and the dry extract could be obtained. To prepare the decaffeinated extract (dGHE), GHE obtained from organic solvent removal was added to 1 N H_2_SO_4_ in a ratio of 5 : 1 (*v*/*v*) and boiled for 3 min. Next, pH was adjusted with NH_4_OH to pH 11 and the alkaline sample was extracted with chloroform through a liquid-liquid partition. This procedure was repeated 3x with 150 mL of chloroform each time. To confirm the absence of caffeine, thin-layer chromatography was used according to Brazilian Pharmacopoeia with Silica Gel GF_254_ as the stationary phase and chloroform : ethanol : formic acid (9 : 0.8 : 0.2, *v*/*v*/*v*) as the eluent and stained with iodine vapors (Figure S1).

### 2.3. Quantitative Analysis of Selected Extract Components

The quantitative analysis of four known components of guarana (caffeine, theobromine, catechin, and epicatechin) was performed by the Instituto Nacional de Pesquisas da Amazônia (INPA) according to the chromatographic method proposed by Sousa et al. [25], with adjustments. Chromatographic analysis was performed with the Shimadzu LC-6A HPLC system, with two LC-6AD pumps, SCL-10AVP controller, SIL-10AF autosampler, and SPD-M20A detector. A 4 × 3 mm ID C18 precolumn (Phenomenex® SecurityGuard™) and a 5 *μ*m, 100 Å, 250 × 4.6 mm ID C18 column (Phenomenex Luna®) were used for the separation. Standard solutions of caffeine, theobromine, catechin, and epicatechin were prepared by diluting the reference standards in methanol and filtering through a PTFE membrane (47 mm, 0.45 *μ*m). The system was calibrated with each reference standard solution at the following range of concentrations: caffeine: 0–380 *μ*g/mL; theobromine: 0–35 *μ*g/mL; and catechin and epicatechin: 0–130 *μ*g/mL. Samples were prepared by diluting the dry GHE extract in methanol at the concentration of 1 mg/mL and filtering it through a PTFE membrane (47 mm, 0.45 *μ*m). The HPLC method conditions were the following: separation method: reverse phase; detection condition: 280 nm; mobile phase: H_2_O : ACN : MeOH : EtOAc (89 mL : 6 mL : 1 mL : 3 mL) + 0.2 mL of HAc; elution: isocratic; and flow rate: 1 mL/min. Calibration curves are shown in Figure S2.

### 2.4. DPPH Analysis

The DPPH radical scavenging activity of GHE was determined as described by Brand-Williams et al. [26]. Briefly, 100 *μ*L of different concentrations of GHE (0 (control), 5, 10, and 50 mg/mL) was added to 3.9 mL of 60 *μ*M DPPH dissolved in 80% methanol. The mixture was homogenized and kept in the dark for 30 min at room temperature. Absorbance of the solution at 515 nm was determined (SP-220 Biospectro, PR, Brazil). A calibration curve was prepared using Trolox ranging in concentration at 200–800 *μ*M. The percentage of inhibition was determined according to %  scavenging activity = (1–Abs  sample  515/Abs  control  515) × 100.

### 2.5. *C. elegans* Maintenance and Treatment with GHE

All nematodes were cultivated on a nematode growth medium (NGM) at 20°C, except for strain CL4176, which were maintained at 16°C. Synchronous L1 populations for experimental procedures were obtained by hypochlorite treatment of gravid hermaphrodites or by egg laying.

Dry GHE extract was diluted in basal solution (0.1 M NaCl, 50 mM KPO_4_ buffer) to a concentration of 50 mg/mL. The suspension was centrifuged at 1500 rpm for 15 min [7], and the supernatant was sterilized by filtration. GHE concentrations of 10 mg/mL and 5 mg/mL were obtained by diluting 50 mg/mL GHE. Basal solution (control) with or without GHE was mixed with an *E. coli* OP50 pellet at OD_600_ = 1 and seeded to NGM plates.

### 2.6. Bioassays for *β*-Amyloid-Induced Paralysis

We used two strains (CL4176 and CL2006) expressing the A*β*1–42 peptide in muscle tissue. A*β* toxicity was assessed by verification of the induced paralytic phenotype. Worms were scored as “paralyzed” based on their incapacity to move their bodies when touched with a platinum loop. CL4176 worms were obtained by synchronous egg laying in NGM plates containing 10 mg/mL or 50 mg/mL GHE or a control solution. Subsequent to an incubation period of 40 h at 16°C, worms were up-shifted from 16°C to 25°C to initiate amyloid-induced paralysis. After 22 h at 25°C, paralysis was scored at 2 h intervals, for 8 h. Each experiment was performed three times with at least 30 worms per group. CL2006 worms were obtained by synchronous egg laying in NGM plates containing either 10 mg/mL or 50 mg/mL GHE or a control solution and incubated for 72 h at 20°C. Paralysis phenotype was accelerated by transferring the worms to 35°C. Paralysis was scored at 1 h intervals for up to 12 h.

RNA interference (RNAi) was performed using the feeding method described previously, with empty pL4440 as controls [27]. Briefly, RNAi clones were grown with 12.5 *μ*g/mL tetracycline and 100 *μ*g/mL ampicillin. On the following day, cultures were diluted in LB supplemented with 60 *μ*g/mL ampicillin and grown to an OD_600_ of 1. This culture was used to seed plates containing 100 *μ*g/mL ampicillin and 1 mM IPTG and left to dry for two days at room temperature. Synchronized L1 larvae were then placed at 20°C on *E. coli* HT115 that expressed target gene RNAi or control RNAi for 46 h, until they reached the L4 stage.

### 2.7. Polyglutamine (PolyQ) Aggregation Quantification

L1 animals from the AM141 strain (*unc-54p::Q40::YFP*) were treated with 10 mg/mL or 50 mg/mL GHE or a control solution for 72 h at 20°C. Next, images of 20 worms from each group were acquired (Axio Imager Z2, Zeiss, NY, USA), and the number of protein aggregates was counted using the software NIH ImageJ software.

### 2.8. Neuronal Survival Assay

L1 animals from the HA759 strain expressing GFP and Htn-Q150 (a polyQ tract of 150 residues derived from the human huntingtin gene) in the ASH neurons were treated with 10 mg/mL or 50 mg/mL GHE or control solution for 6 days at 20°C until adulthood day four. Approximately 70 worms per group were then analyzed with an Axio Imager Z2, Zeiss (NY, USA), to evaluate the fluorescence of bilateral ASH neurons.

### 2.9. Lifespan and Stress Resistance Assays

The lifespan assay was performed with synchronized N2 wild-type animals treated with 10 mg/mL or 50 mg/mL GHE or control solution starting at the L1 stage. The survival analysis consisted of scoring dead/alive animals every day beginning at the first day of adulthood (t0 = day 1) at 25°C. We analyzed approximately 90 animals per group divided into 3 NGM plates. The animals were determined to be dead if no movement was shown with or without stimulation. Animals with hatched eggs internally, extruded parts or those who went missing, were excluded from the data.

Oxidative stress resistance was evaluated in synchronized N2 wild-type animals treated with 10 mg/mL or 50 mg/mL GHE or control solution from the L1 until the L4 stage. L4 animals were then transferred to NGM plates with 10 mM TBHP (*tert-*butyl hydroperoxide) and 12 *μ*g/mL FUDR (fluorodeoxyuridine). Approximately 50 animals per group were analyzed. The survival fractions were scored every three hours until all animals were considered dead, with no pharyngeal pumping or movement. The assay was conducted with five wells per group, each containing approximately ten worms.

Thermotolerance was assessed in animals treated with 10 mg/mL or 50 mg/mL GHE or a control solution from L1 until five-day-old adults at 20°C were transferred onto 3 cm NGM agar plates and incubated at 35°C for 12 h. Survival was monitored at 3 h intervals. Animals that had died from desiccation on the sides of the plate were not included [28].

### 2.10. Measurement of Intracellular ROS

Intracellular ROS was analyzed in N2 and CL2006 strains. Synchronized N2 wild-type animals were treated with 10 mg/mL or 50 mg/mL GHE or control solution for 48 h and transferred to 5 mM H_2_O_2_ for 1 h. Control animals were not treated with H_2_O_2_ and were left in M9 buffer for the same period of time. CL2006-synchronized L1 were treated with 10 mg/mL or 50 mg/mL GHE or control solution for 72 h. Subsequently, 20 to 30 worms per group were collected in PBS + 1% Tween-20, washed twice, and transferred to a 96-well microtiter plate, to which 50 *μ*M H_2_DCF-DA was added. Measurements were performed in triplicate in a multilabel microplate reader (Perkin Elmer Victor X3, MA, USA) at 37°C, with excitation at 485 nm and emission at 535 nm, and the mean values were calculated. Readings were performed every 30 min for 4 h.

### 2.11. Bacterial Growth Curve

*E. coli* OP50 growth was evaluated over 5 h in the presence of basal solution (control) and 10 and 50 mg/mL GHE. OD_600_ readings were performed every 40 min until the control group exceeded OD = 1.00. The OD of the control group at time zero was defined as “value one” against which of all other OD readings were compared.

### 2.12. Analysis of Developmental and Behavioral Parameters

To measure body length, L1 worms of the N2 strain were treated with 10 mg/mL and 50 mg/mL GHE or control solution for 48 h at 20°C, until the L4 stage. Images of 20 worms from each group were acquired (Axio Imager Z2, Zeiss, NY, USA), and body length was measured along the animal axis using AxioVision Rel. 4.8 software.

To assay brood size, 10 L4 worms of the N2 strain were individually transferred to new NGM plates containing either 10 mg/mL or 50 mg/mL GHE or control solution, every day for the duration of the egg laying period. The total progeny numbers for each plate were counted and divided by the number of animals.

Thrash frequency was selected for analysis of locomotion. Worms were treated with 10 mg/mL or 50 mg/mL GHE or control solution for 48 h at 20°C, until the L4 stage. Subsequently, they were individually picked and placed in a drop of M9 and allowed to adapt for 1 min. The number of thrashes was quantified with a stereomicroscope over a 60 sec period. A thrash was defined as a change in the direction of bending at the middle of the body.

Pharyngeal pumping rates were obtained with synchronized N2 animals treated with 10 mg/mL or 50 mg/mL GHE or control solution for 48 h at 20°C since L1 to 1 day of adulthood. The animals were then transferred to NGM plates without bacteria 2 to 3 min before analysis. Pumping rates were assessed in 3 intervals of 20 sec per animal by observing the movements of the terminal bulb of the pharynx. A total of 10 animals from each experimental condition were analyzed.

### 2.13. Reporter Gene Analysis

L1 animals from the transgenic strains CL1553, CL2166, and TJ375 were treated with 10 mg/mL or 50 mg/mL GHE or control solution for 48 h at 20°C, until the L4 stage. Images of 20 worms from each group were acquired (Axio Imager Z2, Zeiss, NY, USA), and fluorescent signals were measured with NIH ImageJ software.

### 2.14. Proteasome Activity Quantification

Approximately 3000 L1 animals from the N2 strain were treated with 10 mg/mL or 50 mg/mL GHE or control solution for 72 h, until 1-day adulthood. Worms were then harvested with M9 buffer and sonicated with a homogenization buffer (5 mM Tris-HCl pH 8.0; 1% glycerol; 1 mM EDTA) plus 10 *μ*L protease inhibitors (Amresco). The lysates were centrifuged at 20000 ×g for 30 min at 4°C. Protein was quantified using the QuantiProTM BCA Assay Kit (Sigma-Aldrich, St. Louis, MO, USA).

Proteasome activity was measured as “chymotrypsin-like activity,” using the peptide substrate succinyl-leu-leu-val-tyr-4-methylcoumaryl-7-amide (SLLVY-MCA) (Sigma-Aldrich, St. Louis, MO, USA). All measurements were performed in the presence and absence of 20 *μ*M MG-132, a proteasome inhibitor. Enzyme kinetics were monitored in a multilabel microplate reader (PerkinElmer VICTOR X3, MA, USA), every 30 minutes for 1 h at 37°C, with excitation at 380 nm and emission at 440 nm. Relative proteasome activity was calculated as the difference between the total activity and the remaining activity in the presence of MG-132.

### 2.15. Lysosomal Quantification

Lysosomal activity was analyzed in strain DA2123 animals, in which the LGG-1 gene is fused with GFP. LGG-1 encodes a protein necessary for the degradation of cellular components through autophagy. Under normal conditions, DA2123 worms exhibit diffuse fluorescence in the cytoplasm of various tissues. The formation of preautophagosomic and autophagosomic structures can be observed and counted with the appearance of focal points of fluorescence that mark autophagic vesicles [29].

### 2.16. Statistical Analyses

All experiments were performed three times. Statistical analyses were performed using GraphPad Prism (v. 5.0) software (CA, USA). Data were analyzed by Kolmogorov-Smirnov test for normality. For normally distributed data, Student's *t-*tests were used to compare groups. Nonparametric data were analyzed by Mann–Whitney test to compare groups. Survival curves were analyzed by the log-rank (Mantel-Cox) test. For all tests, statistical significance was determined as *p* ≤ 0.05.

## 3. Results

### 3.1. GHE Chemical Characterization and Antioxidant Activity *In Vitro*

Methylxanthines and polyphenols in GHE were quantified as previously described in guarana [5, 7] (Table 1): 166.07 *μ*g/mL caffeine, 2.49 *μ*g/mL theobromine, 34.59 *μ*g/mL catechin, and 36.35 *μ*g/mL epicatechin. GHE scavenging potential was measured *in vitro* with a DPPH radical scavenging assay. GHE at 5, 10, and 50 mg/mL had radical scavenging properties equivalent to 500 *μ*M of Trolox, with DPPH's neutralizing capacities varying from 48.30% to 54.37% inhibition. Since the neutralizing capacities of the three concentrations tested were similar, we selected 10 and 50 mg/mL GHE to perform the following assays.

### 3.2. GHE Reduces PolyQ-Mediated Neuronal Death in ASH Neurons and PolyQ Aggregation in Muscle Tissue

**Figure 1:**
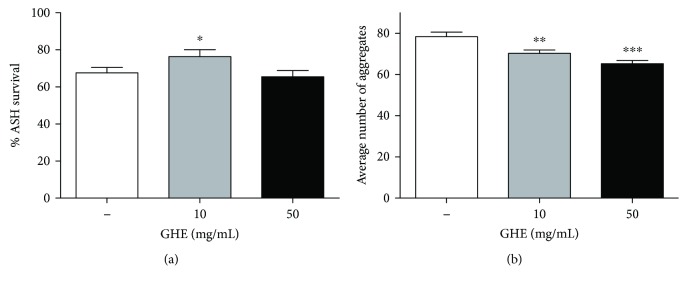
Effect of guarana hydroalcoholic extract (GHE) treatment on polyQ aggregation in *C. elegans* transgenic models of Huntington disease. (a) The *C. elegans* strain HA759 expressing Htn-Q150 in ASH neurons were treated with 10 or 50 mg/mL GHE from L1 until 4-day-old adulthood. Using a fluorescent microscope, ASH neuron death was assessed by loss of bilateral GFP fluorescence. Treatment with 10 mg/mL GHE prevented ASH neuronal death. ^∗^ *p* = 0.048, two-tailed Student *t*-test. (b) The *C. elegans* strain AM141 expressing *polyQ_40_* in muscle cells were treated with either 10 or 50 mg/mL GHE from L1 until 1-day-old adulthood. Images were acquired with a fluorescence microscope, and aggregates were counted. Treatment with either 10 or 50 mg/mL GHE reduced the number of aggregates. ^∗∗^ *p* = 0.004 and ^∗∗∗^ *p* < 0.0001, two-tailed Student *t*-test.

We tested the effect of GHE treatment in HA759 worms that overexpress both GFP and Htn-Q150 primarily in ASH neurons, resulting in its neurodegeneration. In control animals, 67.6% of ASH neurons continued to express GFP at 4-day-old adulthood. On average, the neuronal survival rate significantly increased to 76.4% (*p* = 0.048) on animals treated with 10 mg/mL GHE (Figure 1(a)). In the animals treated with 50 mg/mL GHE, the ASH survival was 65.5%.

We aimed to determine if the effect of GHE in ASH neurons is related to a reduction in protein aggregation. We found that GHE reduced polyQ aggregation in AM141 worms that express *polyQ_40_::YFP* in muscle tissue that shows fluorescent aggregates when the worms reach adulthood. We observed a significant reduction of aggregates after treatment with either 10 or 50 mg/mL (Figure 1(b)). The average number of aggregates observed was 70.29 ± 1.69 (*p* = 0.004) and 65.27 ± 1.57 (*p* < 0.0001) for worms treated with 10 and 50 mg/mL, respectively, compared to 78.45 ± 2.18 aggregates on average for control worms. Similarly, Peixoto et al. [15] found that guarana water extracts reduced polyQ aggregation in muscle. Taken together, these results indicate that GHE treatment reduced polyQ-mediated neuronal death and polyQ aggregation.

### 3.3. GHE Delays A*β*1-42-Induced Paralysis in a SKN-1 and DAF-16-Dependent Manner

**Figure 2:**
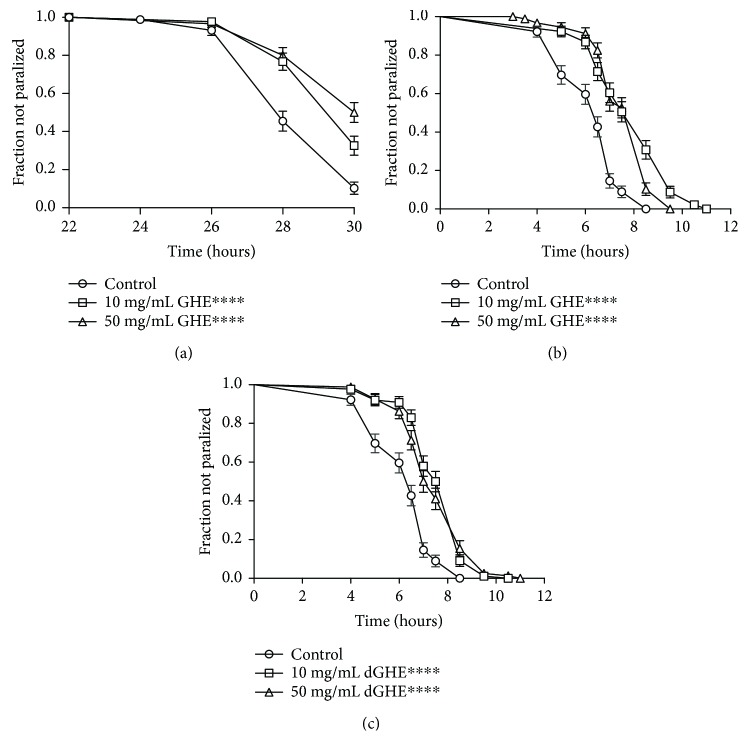
Effect of guarana hydroalcoholic extract (GHE) treatment on the *β*-amyloid induced paralysis in *C. elegans* transgenic models of Alzheimer's disease. Paralysis curves for *C. elegans* strains expressing inducible (CL4176) or constitutive (CL2006) muscle *β*-amyloid treated with either 10 or 50 mg/mL GHE from L1 until 1-day-old adulthood. (a) To initiate *β*-amyloid-induced CL4176, worms were up-shifted from 16°C to 25°C. Paralysis was verified at 2 h intervals following 24 h at 25°C. (b) To accelerate the paralysis, CL2006 worms were transferred to 35°C. Paralysis was verified at 1 h intervals at 35°C. Both strains treated with either 10 or 50 mg/mL GHE showed delayed progression of body paralysis compared to controls. (c) Paralysis curve of CL2006 worms treated with either 10 mg/mL or 50 mg/mL decaffeinated GHE (dGHE), verified at 1 h intervals at 35°C. Both strains treated with either GHE or dGHE showed delayed progression of body paralysis as compared to control worms. ^∗∗∗∗^ *p* < 0.0001 compared to respective controls, log-rank (Mantel-Cox) test.

Next, we assessed the potential beneficial effects of GHE to AD treatment. CL4176 worms are a temperature-sensitive strain that express A*β*1–42 in the body wall muscle at 25°C. Accumulation of A*β*1–42 in muscle cells causes paralysis. We observed a significant delay in paralysis in CL4176 worms fed with 10 and 50 mg/mL GHE (Figure 2(a)). The mean paralysis time for the worms treated with 10 mg/mL and 50 mg/mL GHE was 29.49 (*p* < 0.0001) and 29.53 hours (*p* < 0.0001), an increase of 4.3 and 4.4%, respectively, compared to control worms (Table 2). CL2006 worms that constitutively express A*β*1–42 in the body wall muscle showed a stronger effect of GHE on paralysis time (Figure 2(b)). The mean paralysis time for worms treated with 10 mg/mL and 50 mg/mL GHE was increased by 26.2% (*p* < 0.0001) and 22.3% (*p* < 0.0001), respectively, compared to control worms (Table 2). Similar results were observed when CL2006 worms were treated with decaffeinated GHE (dGHE) (Figure 2(c)). Delay of paralysis was 21.8% with 10 mg/mL dGHE (*p* < 0.0001) and 17.0% with 50 mg/mL dGHE (*p* < 0.0001) compared to control worms (Table 2). These results suggest that GHE treatment is able to delay A*β*1–42-induced paralysis in muscle cells in a caffeine-independent manner.

To explore the possible antiparalysis mechanisms of GHE, we assessed the role of SKN-1, DAF-16, and HSF-1, three major transcription factors involved in stress resistance, longevity, and proteostasis. To this end, we examined the effect of GHE on CL2006 worms using RNAi to knock down *skn-1*, *daf-16*, and *hsf-1* expression. GHE treatment significantly delayed the paralysis rate of CL2006 worms with *hsf-1* RNAi (Table 2). Interestingly, 10 mg/mL GHE treatment failed to delay A*β*1–42-induced paralysis in worms grown on either *skn-1* RNAi (*p* = 0.3500) or *daf-16* RNAi (*p* = 0.4369). However, worms treated with 50 mg/mL GHE on either *skn-1* RNAi or *daf-16* RNAi still showed a significant paralysis delay (*p* < 0.0001) (Table 2). We also knocked down both transcription factors *skn-1* and *daf-16* to assess GHE-induced delayed paralysis (Table 2). Inhibition of both *skn-1* and *daf-16* did not prevent the protective effect of 50 mg/mL GHE treatment on CL2006 worms. Collectively, these results suggest that DAF-16 and SKN-1 are partially required for the protective effect of GHE against A*β*1–42 toxicity.

### 3.4. GHE Increase Lifespan and Thermotolerance

**Figure 3:**
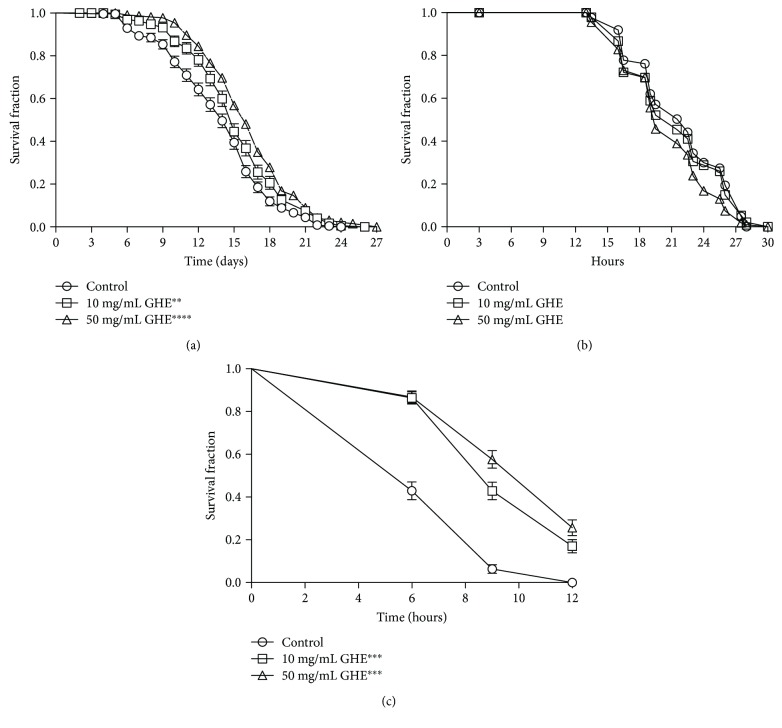
Effect of guarana hydroalcoholic extract (GHE) treatment on *C. elegans* lifespan and stress resistance. (a) Survival curves of wild-type (N2) animals under standard laboratory conditions. Worms were treated with either 10 or 50 mg/mL GHE beginning at L1. Survival was verified every day at 25°C ^∗∗^ *p* = 0.0036 and ^∗∗∗∗^ *p* < 0.0001 compared to respective controls, log-rank (Mantel-Cox) test. (b) Survival curves of wild-type (N2) animals under oxidative stress conditions. Worms were treated with either 10 or 50 mg/mL GHE from L1 until L4 and transferred to plates with 10 mM TBPH. Survival was verified every 3 hours at 20°C. No significant differences were seen between groups, determined by a log-rank (Mantel-Cox) test. (c) Survival curves of wild-type (N2) animals under heat stress. Worms were treated with either 10 or 50 mg/mL GHE from L1 until 5 days of age and incubated at 35°C. The survival was verified 3 h at 35°C. ^∗∗∗^ *p* < 0.0001 compared to respective controls, log-rank (Mantel-Cox) test.

Aging plays an important role in late-onset neurodegeneration [30]. Therefore, we aimed to determine if the effect of GHE on AD and HD models is actually due to a general antiaging effect. First, we determined the lifespan of N2 worms with and without GHE treatment. The lifespan of N2 worms fed GHE significantly increased compared to control worms (Figure 3(a)). The mean lifespan of worms fed without GHE was 13.91 ± 0.26 days, while worms fed 10 and 50 mg/mL GHE had a mean lifespan of 15.25 ± 0.29 (*p* = 0.0036) and 16.30 ± 0.26 days (*p* < 0.0001), which represents a 9.6 and 17.1% increase compared to control worms, respectively (Table 3).

Several studies have shown that lifespan extension is closely associated with enhanced resistance to various forms of environmental stressors [31]. Thus, we assessed the effects of GHE treatment on resistance to oxidative and heat stress in N2 worms. To assess oxidative stress resistance, we compared the survival rate of N2 worms treated with either 10 or 50 mg/mL GHE to untreated worms exposed to 10 mM TBHP. Treatment with 10 mg/mL GHE did not significantly increase oxidative stress resistance compared to controls (Figure 3(b)). The mean survival time for the animals treated with 10 mg/mL GHE was 21.75 ± 0.38 hours compared to 21.32 ± 0.39 to controls (*p* = 0.4631) (Table 3). The mean survival time for the animals treated with 50 mg/mL was 20.55 ± 0.35 hours, which represents a significant reduction of −5.5% survival time during oxidative stress when compared to controls (*p* = 0.0092) (Table 3). The effect of GHE on heat tolerance was analyzed at 35°C in five-day-old N2 worms treated with either 10 or 50 mg/mL GHE. We observed that animals treated with either 10 or 50 mg/mL GHE showed increased thermotolerance compared to controls (Figure 3(c)). The mean survival time for the untreated animals was 7.47 ± 0.15 hours. Worms treated with 10 mg/mL GHE displayed a 32.1% extension of mean survival to 9.87 ± 0.17 hours (*p* < 0.0001), whereas worms treated with 50 mg/mL GHE displayed a 38.3% extension of mean survival to 10.33 ± 0.18 hours (*p* < 0.0001) (Table 3).

### 3.5. The Protective Effects of GHE Are Not Related to Antimicrobial Effects

**Figure 4:**
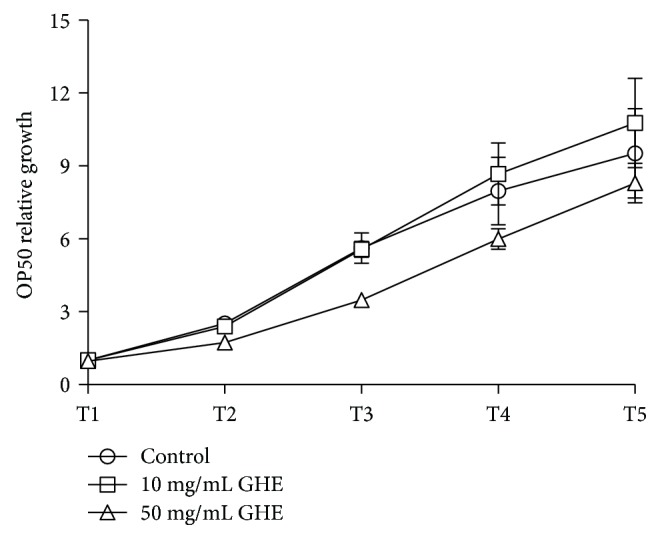
Effects of guarana hydroalcoholic extract (GHE) on bacterial growth. LB media supplemented with either 10 or 50 mg/mL GHE was used for bacterial growth. *Escherichia coli* OP50 growth was monitored by measuring absorbance at 600 nm for 5 h. Data are presented as mean ± SD (*n* = 3). All OD readings at 600 nm were normalized to the OD of controls at time zero. No significant differences between groups were observed at any time point, as determined by a two-tailed Student *t*-test.

It has been shown that differences in bacterial strains, such as food source, can influence longevity and even toxicity and aggregation phenotypes in *C. elegans* neurodegenerative models [32–34]. Since guarana has been reported to have antimicrobial properties [6, 35], we tested whether the protective effect of GHE was the result of bacterial growth inhibition. We monitored the growth of *E. coli* OP50 over 5 h in the presence of GHE and did not observe an antimicrobial effect of GHE at either concentration (Figure 4).

### 3.6. GHE Treatment Increases Metabolism without Interfering with Reproductive Output

**Figure 5:**
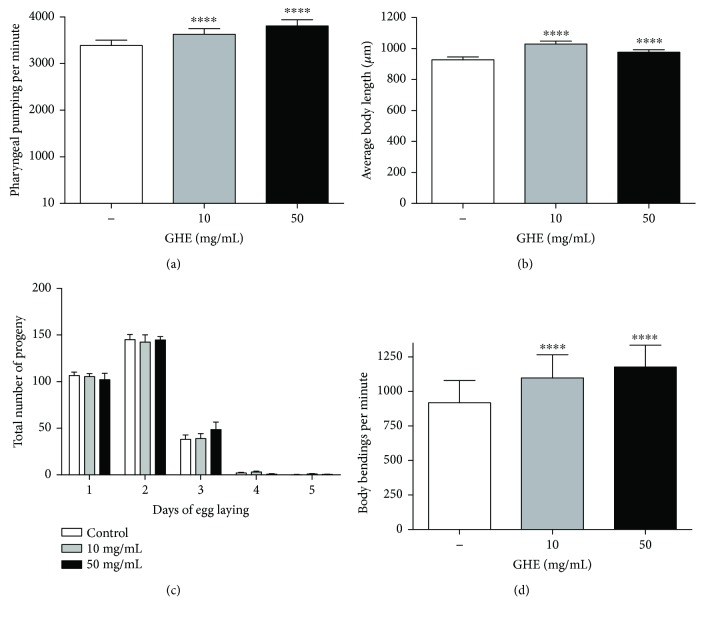
Effect of guarana hydroalcoholic extract (GHE) treatment on growth, reproduction, feeding, and locomotion. (a) Pharyngeal pumping rate was quantified in L4 wild-type (N2) animals treated with either 10 or 50 mg/mL GHE. ^∗∗∗∗^ *p* < 0.0001, two-tailed Student *t*-test. (b) *C. elegans* growth was measured in 1-day-old animals treated with either 10 or 50 mg/mL GHE from L1. Images were captured from each animal, and body length was measured along the animal axis using NIH ImageJ software. ^∗∗∗∗^ *p* < 0.0001, two-tailed Student *t*-test. (c) Reproduction was measured in wild-type (N2) animals treated or not with either 10 mg/mL GHE and 50 mg/mL GHE from L1. Animals were individually transferred daily to new plates until the end of the reproductive period. (d) Body bend frequency was measured in L4 animals treated with either 10 or 50 mg/mL GHE from L1. ^∗∗∗∗^ *p* < 0.0001, two-tailed Student *t*-test.

An organism's longevity can be affected by food intake [36] and reproductive output [37]. To determine if GHE would reduce food intake and, thus, reduce calorie intake, we measured pharyngeal pumping in N2 worms. The pharyngeal pumping rate of worms fed with either 10 or 50 mg/mL GHE was significantly increased (Figure 5(a)), suggesting that the effects of GHE are not due to calorie restriction. The increased pharyngeal pumping rate observed in worms treated with GHE was also associated with increased body size (Figure 5(b)), suggesting that GHE treatment does not reduce food intake. To test whether GHE treatment could interfere with reproductive capacity, we measured the total number of progeny. No change in brood size was observed after treatment with either 10 or 50 mg/mL GHE (Figure 5(c)).

Since locomotor behavior of *C. elegans* decreases with age, we investigated whether GHE alters motility. We monitored the body bends of worms treated with GHE at the L4 stage. As shown in Figure 5(d), treatment with GHE significantly increased the frequency of body bends. Taken together, these results suggest that GHE treatment increases metabolism without interfering with reproductive output.

### 3.7. GHE Reduces ROS Accumulation

**Figure 6:**
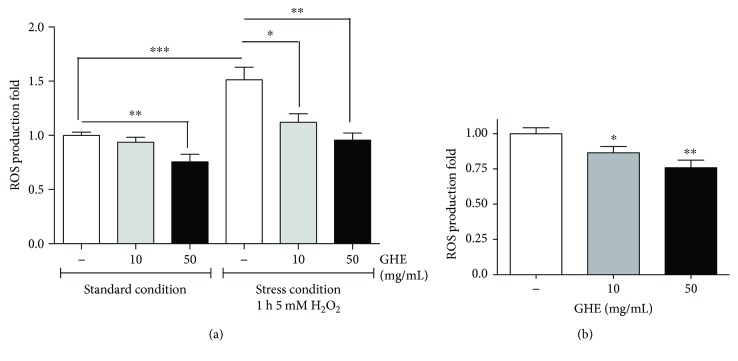
Effect of guarana hydroalcoholic extract (GHE) treatment on intracellular ROS accumulation in *C. elegans.* (a) Wild-type and (b) CL2006 animals were treated with either 10 or 50 mg/mL GHE from L1 until L4. For wild-type animals, ROS production was evaluated on both standard and stress conditions induced by 5 mM H_2_O_2_ for 1 h. ROS levels were measured using the dye H_2_DCFDA. Results are expressed as H_2_DCFDA fluorescence relative to untreated controls. ^∗^ *p* < 0.05, ^∗∗^ *p* < 0.007, and ^∗∗∗^ *p* = 0.0006, two-tailed Student *t*-test.

To investigate the mechanism underlying GHE-induced A*β* toxicity attenuation, lifespan enhancement, and thermotolerance in *C. elegans*, we evaluated the effect of GHE on the intracellular accumulation of ROS in both N2 and CL2006 worms. In N2 worms, 50 mg/mL GHE treatment significantly decreased ROS production under standard conditions, and both 10 and 50 mg/mL GHE reduced ROS production under stress conditions (Figure 6(a)). In CL2006 worms, ROS production was significantly reduced after GHE treatment with either 10 mg/mL (*p* = 0.0440) or 50 mg/mL (*p* < 0.01) (Figure 6(b)). Therefore, it is likely that the antiparalysis and pro-longevity effect of GHE may be, in part, due to the reduction of oxidative stress and ROS levels.

### 3.8. GHE Induces the Expression of Antioxidant and Heat Shock-Associated Genes

**Figure 7:**
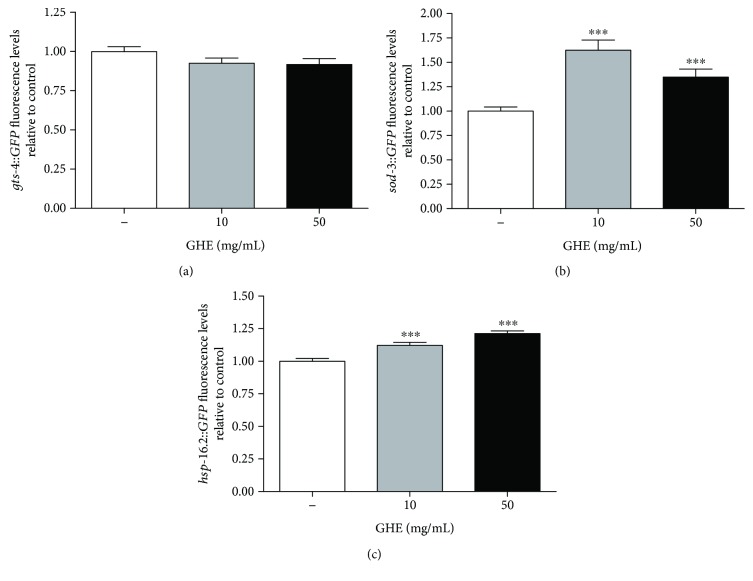
Effect of guarana hydroalcoholic extract (GHE) treatment on antioxidant and chaperonin gene expression. (a) Analysis of *sod-3::GFP*, (b) *gst-4::GFP*, and (c) *hsp-16.2::GFP*. Transgenic worms were treated with either 10 or 50 mg/mL GHE for 48 h beginning at L1. Images were acquired on a fluorescence microscope, and GFP fluorescence signals were measured using NIH ImageJ software. ^∗∗∗^ *p* < 0.0001, two-tailed Student *t*-test.

To determine the molecular responses associated with the protective effects of GHE in *C. elegans*, we analyzed the gene expression of three reporter genes related to stress resistance and longevity. We selected glutathione-s-transferase-4 (*gst-4*), a SKN-1 target gene [38], and superoxide dismutase 3 (*sod-3*) and heat-shock protein 16.2 (*hsp-16.2*), two known DAF-16 target genes [39]. The fluorescence signals of *gst-4::GFP* worms treated with either 10 or 50 mg/mL of GHE were not significantly different compared to controls (Figure 7(a)). The expression of *sod-3::GFP* and *hsp-16.2::GFP* increased after GHE treatment with either 10 or 50 mg/mL compared to untreated worms (Figures 7(b) and 7(c)). These results suggest that GHE treatment might increase longevity and thermotolerance by activating DAF-16 target genes.

### 3.9. GHE Increases Proteasomal and Lysosomal Degradation

**Figure 8:**
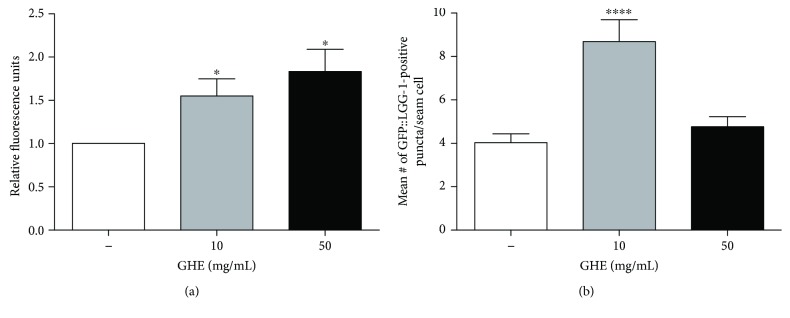
Effect of guarana hydroalcoholic extract (GHE) treatment on protein degradation activity and lysosome-related organelle levels (or protein homeostasis). (a) Animals were treated with either 10 or 50 mg/mL GHE from L1 until L4. Proteasome chymotrypsin-like activity was monitored by SLLVY-MCA digestion in worm extracts containing equal amounts of total protein. ^∗^ *p* < 0.05, two-tailed Student *t*-test. (b) *GFP::LGG-1* animals were treated with either 10 or 50 mg/mL GHE from L1 until L4. *GFP::LGG-1* puncta were counted in seam cells to quantify the mean number of puncta per seam cell. ^∗∗∗∗^ *p* < 0.0001, two-tailed Student *t*-test.

Alterations in protein homeostasis underlie the etiology of many neurodegenerative diseases [40]. To evaluate whether GHE treatment alters protein homeostasis, we measured the proteasome and lysosome activity in animals treated with GHE. Proteasome chymotrypsin-like activity was monitored by SLLVY-MCA digestion in L4 worm extracts containing equal amounts of total protein. GHE treatment with 10 and 50 mg/mL increased proteasome degradation activity relative to controls by 50% (*p* = 0.0496) and 80% (*p* = 0.0305), respectively (Figure 8(a)). Autophagy activity was evaluated by quantifying the number of GFP-positive foci in the seam cells of the *GFP::LGG-1* transgene. LGG-1 encodes a protein necessary for the degradation of cellular components by autophagy. Under normal conditions, DA2123 worms exhibit diffuse fluorescence in the cytoplasm of various tissues. The formation of preautophagosomic and autophagosomic structures can be observed and counted with the appearance of focal points of fluorescence (puncta) [29]. Autophagy was increased only in worms treated with 10 mg/mL GHE (*p* < 0.0001) (Figure 8(b)).

## 4. Discussion

Previous studies have indicated that guarana has some protective effects in PD [13], AD [5], and HD models [15]. However, little is known regarding the mechanisms underlying this effect. Here, we show that treatment with GHE reduced polyQ aggregation in muscles, reduced polyQ-mediated neuronal death in ASH neurons, and delayed A*β*1–42-induced paralysis. Genetic analyses using the transgenic strain CL2006 suggest that the GHE antiparalysis effect is partially dependent on the DAF-16 and SKN-1 transcription factors. In accordance with this, we show that the protective effect of GHE against aggregation-prone diseases is associated with antioxidant activity and modulation of proteostasis. We observed increased lifespan and proteasome activity, reduced intracellular ROS, and autophagosome accumulation in transgenic lines. Additionally, GHE increased the expression of the antioxidant enzyme SOD-3 and the chaperonin HSP-16.2, which are well-known effector genes regulated by DAF-16.

The presence of toxic and insoluble protein aggregates is a common feature of more than 20 neurodegenerative conditions [40–42]. For example, HD is an autosomal-dominant neurodegenerative disorder with earlier onset, as its first symptoms may appear at age 40 [43]. HD is a polyglutamine disease, genetically characterized by glutamine (CAG) repetitions on huntingtin (Htn) protein gene [43]. The proteolytic cleavage of Htn results in the formation of polyQ oligomers, aggregates, and inclusions [44]. AD is a chronic and multifactorial neurodegenerative disorder, mostly later onset, with first symptoms appearing after age 65 [45, 46]. AD physiopathology is characterized by the presence of three common characteristics: the presence of amyloid-*β* (A*β*) peptide aggregates, known as senile plaques; the formation of neurofibrillary tangles, composed by TAU protein; and progressive neurodegeneration [45, 47].

Previous studies have shown that guarana reduced A*β* peptide aggregation *in vitro* [5] and polyQ aggregation in *C. elegans* [15]. Our results show that both concentrations of GHE studied here reduced protein aggregation in AM141 worms, which express polyQ40 fragments in the muscle, replicating the results of Peixoto et al. [15] that used the same strain with a guarana water extract. The capacity of GHE to reduce protein-associated toxicity was also observed in both AD models evaluated in this study. Specifically, GHE treatment resulted in delayed onset of paralysis induced by A*β* deposits in muscle cells. Hence, we show that GHE may reduce protein aggregation and protect the correlated toxicity. However, to evaluate if GHE could effectively protect against neurodegeneration and neuronal cell death, we tested whether GHE has beneficial effects in the *C. elegans* strain expressing Htn-Q150 in the ASH neuron. Our results show that 10 mg/mL GHE has a neuroprotective effect, since it reduced the death of ASH neurons expressing Q150 fragments. Thus, the neuroprotection effect of GHE suggests that it may be useful as a therapy for neurodegenerative diseases associated with aggregation-prone proteins.

Caffeine is well known as the major constituent of guarana [5, 7]. It has been demonstrated that chronic and moderate consumption of caffeine affects the pathophysiology of neurodegenerative diseases [4]. Studies in *C. elegans* have demonstrated the positive impact of caffeine and coffee extract in reducing protein aggregation toxicity in both HD and AD models [24, 48, 49]. Despite the high content of caffeine in GHE, we found that its protective effect against A*β*1–42 toxicity in muscle cells is not exclusively dependent on the presence of caffeine, since decaffeinated GHE also delayed A*β*-induced paralysis. Similar results were observed by Dostal et al. [24] with a coffee extract. They observed that when using decaffeinated coffee, the extract was still able to delay the paralysis onset in *C. elegans*. These results suggest a synergic effect of all of the GHE phytochemical constituents. As shown in this study, besides methylxanthines such as caffeine, GHE also has a considerable number of polyphenol constituents, which may explain the beneficial effects of GHE.

Previous studies reported that DAF-16, SKN-1, and HSF-1 play pivotal roles in regulating longevity and ameliorating A*β* and polyQ aggregation and toxicity [21, 22, 24, 48]. Our results showed that RNAi of HSF-1 did not abolish GHE-mediated delay of body paralysis, whereas RNAi of DAF-16 and SKN-1 eliminated the delay of paralysis progression at the lower treatment dose used here (10 mg/mL). These results suggest that the protection provided by GHE against A*β*1–42 toxicity is partially dependent on DAF-16 and SKN-1. Since both transcription factors are key regulators of many important biological processes, including lifespan, stress responses, and proteostasis, we reasoned that GHE treatment might protect worms against polyQ and A*β*1–42 toxicity by increasing antioxidant capacity and proteostasis.

Despite that oxidative damage to neurons may not be the primary event initiating neurodegenerative disorders, it seems that oxidative stress participates in the pathogenetic cascade of these diseases [50]. Our results are consistent with previous studies that show that guarana presents *in vitro* antioxidant capacity [7–9, 51]. Therefore, we investigated if the protective effects of GHE were related to the *C. elegans* redox status.

Our results show that GHE treatment effectively reduced intracellular ROS levels in N2 worms (wild-type) under standard and stress conditions. In CL2006 worms, GHE treatment reduced the increased levels of ROS levels induced by the internal expression of A*β* seen in this strain [52]. GHE's ability to influence the redox state of the organism was positively related to an increase in lifespan under standard and not under oxidative stress conditions. The antiaging effect of guarana under standard conditions has been previously described by Peixoto et al. [15] using a guarana water extract. Interestingly, Peixoto et al. [15] observed an increase in survival rate of animals treated with guarana under oxidative stress. This dissimilarity may be explained by the differences in extract preparation; we used a hydroalcoholic extract, whereas Peixoto et al. [15] used a water-based extraction method, which may have changed the phytochemical components obtained in the extract. Moreover, we examined the effect of GHE on *sod-3* and *gst-4*. Superoxide dismutase *sod-3* is a DAF-16 target gene, which has an important role in protecting against ROS. *gst-4* encodes for a phase II detoxification enzyme glutathione-S-transferase regulated by SKN-1. Consistent with Peixoto et al. [15], we observed that GHE treatment increased the expression of *sod-3::GFP.* Considering the role of GST-4 in xenobiotic metabolism [53], our result suggests that GHE is not toxic in *C. elegans*. GHE treatment increased the expression of *sod-3::GFP*, suggesting that GHE activates enzymes that help attenuate the oxidative stress generated by H_2_O_2_ and the presence of amyloid-*β* peptide.

Proteostasis collapse leads to impaired protein solubility and increased cytotoxicity, which are hallmark characteristics of aging and neurodegenerative diseases [54]. Increased proteasome activity and chaperone activation are among the highly conserved responses retained by eukaryotic cells to maintain proteostasis. Previous reports showed that *in vivo β*-amyloid peptide toxicity was suppressed by overexpression of HSP-16.2 in *C. elegans* [23]. Here we show that GHE increased the expression of chaperonin *hsp-16.2* under standard and heat shock conditions (Figure S3). Since heat stress is known to generate protein damage, the increased *hsp-16.2* expression may also explain the increased survival rate of GHE-treated worms at 35°C. Moreover, GHE treatment resulted in doubled proteasome activity and increased autophagosome formation. These results suggest that GHE treatment protects worms against polyQ and A*β*1–42 toxicity, in part, by increasing the expression of heat shock protein-related genes and increasing protein degradation activity.

Although the effects of GHE on oxidative stress responses and proteostasis were directly observed in this study, we attempted to exclude other causes of the beneficial effects of GHE, including antimicrobial effects, caloric restriction, or developmental impairment. It is well established that the bacteria used as a food source to *C. elegans* might be pathogenic, and, therefore, some substances might be beneficial merely due its antimicrobial effects [55]. Even though previous studies have shown that guarana extracts present antibacterial properties [6, 35], in our study, GHE had no effect on *E. coli* growth. Furthermore, no significant difference on reproduction or body length was detected in worms treated with GHE, suggesting that the energy required for life extension was not obtained from reproduction or growth rate impairments. Conversely, we observed an increase in the body bending frequency in worms exposed to GHE. Finally, the pharyngeal pumping rate was significantly higher in worms treated with GHE, excluding any caloric mechanism associated with GHE. Interestingly, animals treated with GHE showed decreased intestinal fat levels as visualized by Nile Red staining (data not shown). Taken together, these results suggest that GHE treatment has a stimulant effect that increases metabolism without interfering with development or reproductive output.

## 5. Conclusions

**Figure 9:**
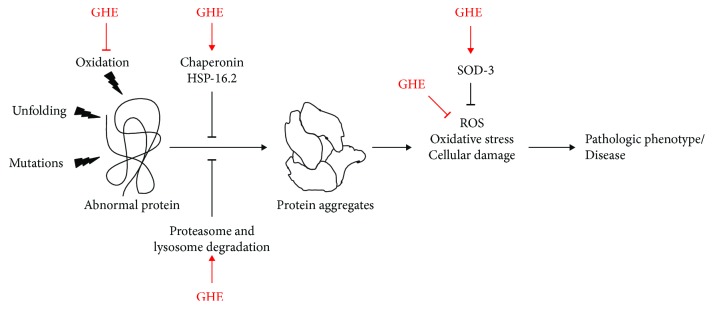
Hypothetical model of the mode of action of guarana hydroalcoholic extract (GHE) on *C. elegans*. Abnormal proteins are formed due to oxidation, misfolding, or mutations in specific genes. Cellular protein quality control systems, such as chaperones, proteasomes, and lysosomes, act to repair or degrade the abnormal proteins. These proteins, when not repaired or degraded, can be aggregated into cells, which increase endogenous ROS level, oxidative stress, and cellular damage. Our results indicate that GHE treatment is able to reduce the pathological phenotypes associated with AD and HD in *C. elegans* through the induction of proteasome and lysosome activity and the expression of chaperone HSP-16.2. GHE treatment also reduces oxidative stress by neutralizing ROS and inducing the expression of an antioxidant enzyme, SOD-3.

This study demonstrated the GHE-induced protection of *C. elegans* in AD and HD models, by the activation of antioxidant and protein degradation pathways (Figure 9). Although the beneficial effects of GHE have previously been studied in various models, including humans, our study is the first to investigate the genetic requirements associated with the GHE-mediated anti-AD effects. Our findings reveal that two highly conserved transcription factors, DAF-16 and SKN-1, are partially responsible for this beneficial effect against A*β* toxicity. Even though this dependency was partial, neither caloric restriction and antimicrobial effects nor development and reproduction impairment influenced the action of GHE. Finally, GHE prevented the death of ASH neurons overexpressing human Htn150 suggesting its therapeutic potential in combating age-related diseases.

## Figures and Tables

**Table 1:** Quantitative phytochemicals in guarana hydroalcoholic extract (GHE).

Compound	Concentration (*μ*g/mL)
Methylxanthines	
Caffeine	166.07 ± 10.61
Theobromine	2.48 ± 0.62
Polyphenols	
Catechins	34.59 ± 10.11
Epicatechins	36.25 ± 9.65

**Table 2:** Effect of guarana hydroalcoholic extract (GHE) treatment on *β*-amyloid induced paralysis of CL4176 and CL2006 strains.

Strains and condition	Mean paralysis time (hours ± SEM)	% mean paralysis time variation versus control	*p* value (log rank) versus control^a^	*N* ^b^
CL4176 on *E. coli* OP50				
Control	28.27 ± 0.38			79 (3)
10 mg/mL GHE	29.49 ± 0.10	4.3	<0.0001	58 (3)
50 mg/mL GHE	29.53 ± 0.10	4.4	<0.0001	45 (3)
CL2006 on *E. coli* OP50				
Control	6.28 ± 0.13			
10 mg/mL GHE	7.93 ± 0.16	26.2	<0.0001	91 (0)
50 mg/mL GHE	7.68 ± 0.13	22.3	<0.0001	92 (3)
10 mg/mL dGHE	7.65 ± 0.13	21.8	<0.0001	88 (0)
50 mg/mL dGHE	7.41 ± 0.14	17.0	<0.0001	80 (1)
CL2006 on *control(RNAi)*				
Control	7.39 ± 0.13			
10 mg/mL GHE	8.56 ± 0.14	15.8	<0.0001	89 (2)
50 mg/mL GHE	9.09 ± 0.13	23.0	<0.0001	90 (1)
CL2006 on *hsf-1(RNAi)*				
Control	8.12 ± 0.14			85 (3)
10 mg/mL GHE	9.24 ± 0.12	13.8	<0.0001	95 (3)
50 mg/mL GHE	9.22 ± 0.14	13.5	<0.0001	87 (3)
CL2006 on *daf-16(RNAi)*				
Control	6.82 ± 0.13			91 (3)
10 mg/mL GHE	6.90 ± 0.15	1.2	0.4369	89 (3)
50 mg/mL GHE	7.74 ± 0.14	13.5	<0.0001	90 (3)
CL2006 on *skn-1(RNAi)*				
Control	6.94 ± 0.28			78 (3)
10 mg/mL GHE	7.21 ± 0.27	3.5	0.3500	73 (3)
50 mg/mL GHE	8.16 ± 0.23	17.6	0.0021	79 (3)
CL2006 on *skn-1(RNAi);daf-16(RNAi)*				
Control	7.16 ± 0.12			87 (3)
10 mg/mL GHE	7.34 ± 0,12	3.2	0.3647	92 (3)
50 mg/mL GHE	8.07 ± 0.15	12.7	<0.0001	88 (4)

^a^Comparisons were performed using log-rank (Mantel-Cox) test. ^b^Total number of animals analyzed. The number in parentheses indicates the number of independent trials.

**Table 3:** Effect of guarana hydroalcoholic extract (GHE) treatment on survival of wild-type animals on standard and stress conditions.

Condition	Mean survival^a,b^	% mean survival time variation versus control	*p* value (log rank) versus control^c^	*N* ^d^
Standard^a^				
Control	13.91 ± 0.26			238 (3)
10 mg/mL GHE	15.25 ± 0.29	9.6	0.0036	181 (3)
50 mg/mL GHE	16.30 ± 0.26	17.1	<0.0001	199 (3)
Oxidative stress (10 mM TBHP)^b^				
Control	21.75 ± 0.38			105 (3)
10 mg/mL GHE	21.32 ± 0.39	−2.0	0.4631	115 (3)
50 mg/mL GHE	20.55 ± 0.35	−5.5	0.0092	118 (3)
Heat stress (35°C)^b^				
Control	7.47 ± 0.15			142 (3)
10 mg/mL GHE	9.87 ± 0.17	32.1	<0.0001	122 (3)
50 mg/mL GHE	10.33 ± 0.18	38.3	<0.0001	107 (3)

^a^Survival time measured in days ± SEM. ^b^Survival time measured in hours ± SEM. ^c^Comparisons were performed using log-rank (Mantel-Cox) test. ^d^Total number of animals analyzed. The number in parentheses indicates the number of independent trials. TBHP: *tert-*butyl hydroperoxide.

## Data Availability

Anyone interested to access the data of this study can contact the corresponding author at rivaoliveira@cb.ufrn.br.

## Supplementary Materials

Supplementary Materials Figure S1: thin layer chromatography profile of the decaffeinated guarana hydroalcoholic extract (dGHE). Figure S2: quantitative analysis of selected components of the extract. Figure S3: effect of guarana hydroalcoholic extract (GHE) treatment on *hsp-16.2::GFP* gene expression under heat shock condition.
