# Supplementary material for: Guarana (Paullinia cupana) Extract Protects Caenorhabditis elegans Models for Alzheimer Disease and Huntington Disease through Activation of Antioxidant and Protein Degradation Pathways
Source: Oxid Med Cell Longev. 2018 Jul 4;2018:9241308. doi: 10.1155/2018/9241308 (PMC6079341; doi:10.1155/2018/9241308)
Supplement: Supplementary Materials — Figure S1: thin layer chromatography profile of the decaffeinated guarana hydroalcoholic extract (dGHE). Figure S2: quantitative analysis of selected components of the extract. Figure S3: effect of guarana hydroalcoholic extract (GHE) treatment on hsp-16.2::GFP gene expression under heat shock condition. [file 9241308.f1.pdf]

Guarana (*Paullinia cupana*) Extract Protects *Caenorhabditis elegans* Models for Alzheimer Disease and Huntington Disease through Activation of Antioxidant and Protein Degradation Pathways

Patrícia Ferreira Boasquívis et al.

Supplementary Material

Material and Methods

Preparation of decaffeinated Guarana Hydro-alcoholic Extract (dGHE)

To prepare the decaffeinated extract (dGHE), GHE obtained from organic solvent removal was added to 1N H_2SO_4 in a ratio of 5:1 (v/v) and boiled for 3 min. Next, pH was adjusted with NH_4OH to pH 11 and the alkaline sample was extracted with chloroform through a liquid-liquid partition. This procedure was repeated 3x with 150 mL of chloroform each time. To confirm the absence of caffeine, Thin Layer Chromatography was used according to Brazilian Pharmacopeia with Silica Gel GF_{254} as the stationary phase, chloroform: ethanol: formic acid (9:0.8:0.2, v/v/v) as the eluent, and stained with iodine vapors (Figure S1).

Quantitative Analysis of Selected Extract Components

The quantitative analysis of four known components of Guarana (caffeine, theobromine, catechin and epicatechin) was performed by the Instituto Nacional de Pesquisas da Amazônia (INPA) according to the chromatographic method proposed by Souza et al (2010), with adjustments. Chromatographic analysis was performed with the Shimadzu LC-6A HPLC system, with two LC-6AD pumps, SCL-10Avp controller, SIL-10AF auto-sampler and SPD-M20A detector. A 4 x 3 mm I.D. C18 pre-column (Phenomenex® SecurityGuard™) and a 5 µm, 100 Å, 250 x 4,6 mm I.D. C18 column (Phenomenex® Luna®) were used for the separation. Standard solutions of caffeine, theobromine, catechin and epicatechin were prepared by diluting the

reference standards in methanol and filtering through a PTFE membrane (47 mm, 0,45 µm). The system was calibrated with each reference standard solution at the following range of concentrations: caffeine: 0 - 380 µg/mL; theobromine: 0 - 35 µg/mL; catechin and epicatechin: 0 - 130 µg/mL. Samples were prepared by diluting dry GHE extract in methanol at concentration of 1 mg/mL and filtering it through a PTFE membrane (47 mm, 0,45 µm). The HPLC method conditions were: separation method: reverse phase; detection condition: 280 nm; mobile phase: H_2O:ACN:MeOH:AcOEt (89 mL: 6 mL: 1 mL: 3 mL) + 0,2 mL de HAc; elution: isocratic; flow rate: 1 mL/min. Calibration curves are shown in supplementary Figure S2.

Analysis of *hsp-16.2::GFP* Expression

The transgenic strains *hsp-16.2::GFP* were treated or not with GHE and then transferred or not to 35°C for 1 h followed by a 1 h and 30 min recovery period. Twenty worms from each group were photographed on a fluorescence microscope (Zeiss Axio Imager Z2, NY, USA) using a 10x ocular lens. GFP fluorescence signals were measured using NIH Image J software. Three experiments were performed.

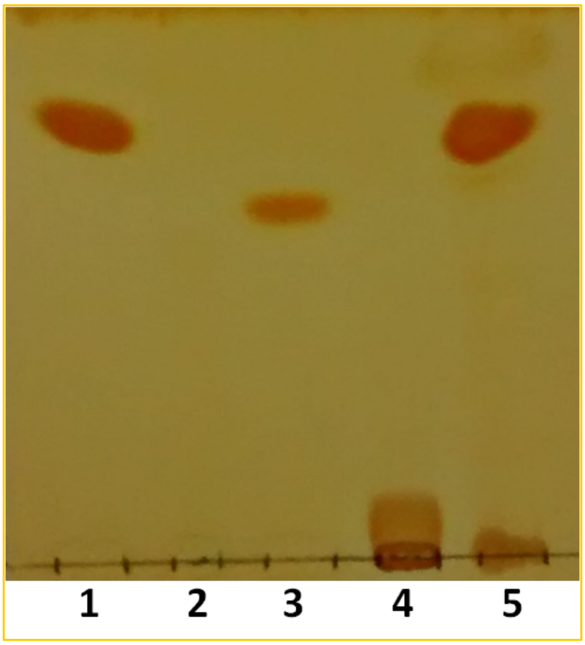

Figure S1: Thin layer chromatography profile of the decaffeinated guarana hydroalcoholic extract (dGHE).

Samples loaded were caffeine (1), theobromine (2), theophylline (3), dGHE (4) and chloroform fraction obtained by liquid-liquid extraction at pH 11 (5).

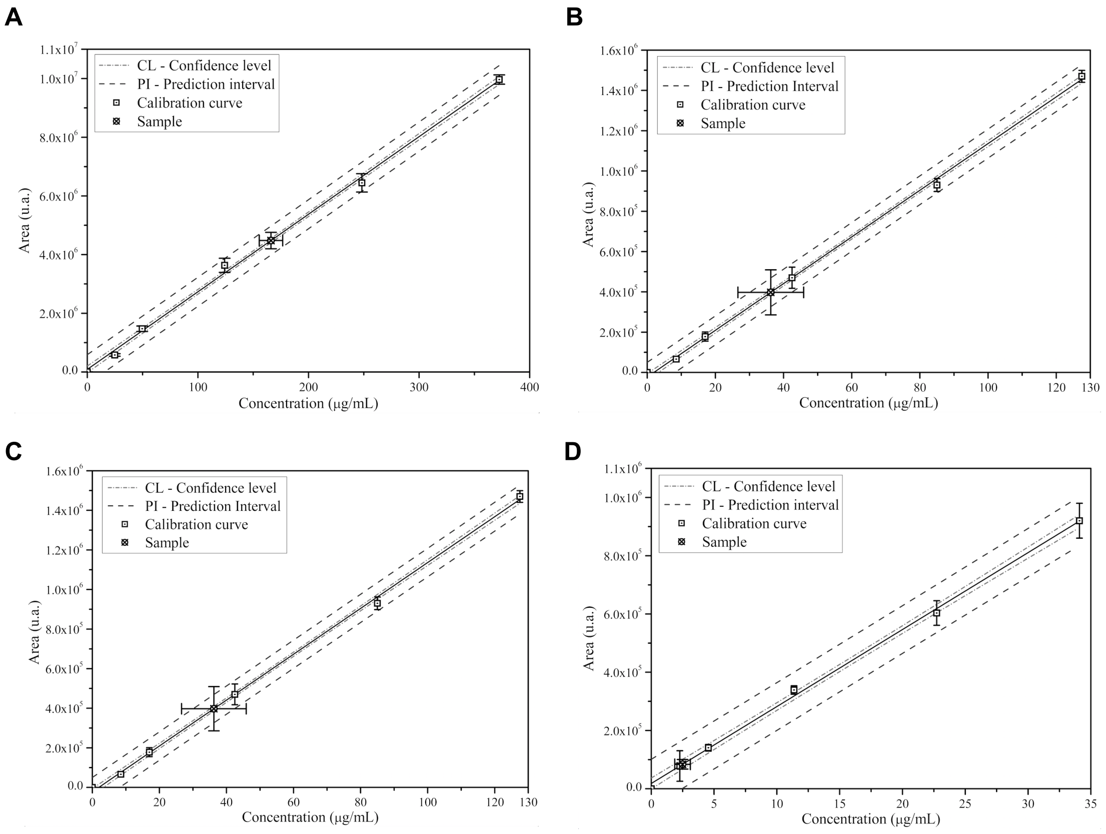

Figura S2 - Quantitative analysis of selected components of the extract

Calibration curve of caffeine (A), catechin (B), epicatechin (C) and theobromine (D) standards.

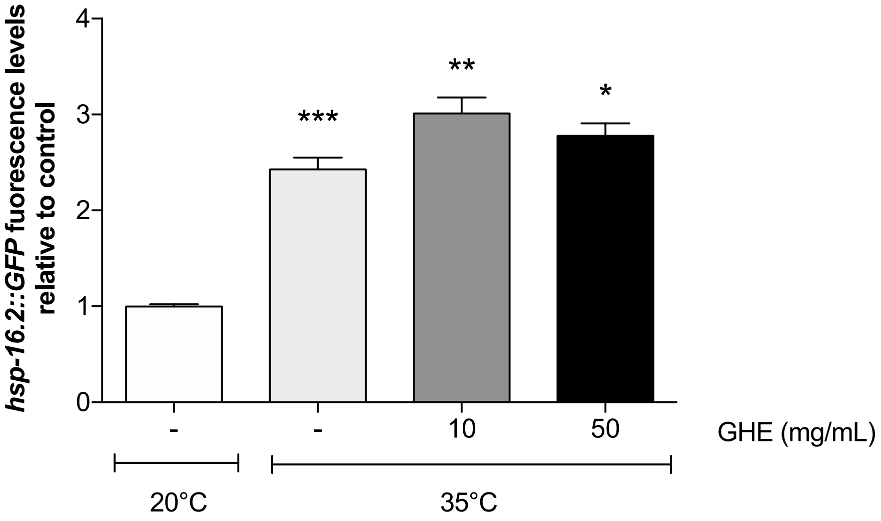

Figure S3 – Effect of guarana hydroalcoholic extract (GHE) treatment on *hsp-16.2::GFP* gene expression under heat shock condition

Transgenic worms were treated with either 10 or 50 mg/mL GHE for 48 h beginning at L1 and then submitted to heat shock at 35°C for 1 h. Photographs were taken on a fluorescence microscope and GFP fluorescence signals were measured using NIH Image J software. *** $p<0.0001$ compared to control at 20°C and **$p = 0.006$ and * $p=0.0456$ compared to control at 35°C by a two-tailed Student's t-test.
